# Characterization of resting state activity in MCI individuals

**DOI:** 10.7717/peerj.135

**Published:** 2013-08-20

**Authors:** Roberto Esposito, Alessandra Mosca, Valentina Pieramico, Filippo Cieri, Nicoletta Cera, Stefano L. Sensi

**Affiliations:** 1 Department of Neuroscience and Imaging, University “G. d’Annunzio” Chieti-Pescara, Chieti, Italy; 2 Molecular Neurology Unit, Center of Excellence on Aging, University “G. d’Annunzio”, Chieti-Pescara, Chieti, Italy; 3 Departments of Neurology and Pharmacology, Institute for Memory Impairments and Neurological Disorders, University of California-Irvine, Irvine, CA, USA

**Keywords:** rs-fMRI, MCI, Aging, AD, Alzheimer

## Abstract

**Objectives.** Aging is the major risk factor for Alzheimer Disease (AD) and Mild Cognitive Impairment (MCI). The aim of this study was to identify novel modifications of brain functional connectivity in MCI patients. MCI individuals were compared to healthy elderly subjects.

**Methods.** We enrolled 37 subjects (age range 60–80 y.o.). Of these, 13 subjects were affected by MCI and 24 were age-matched healthy elderly control (HC). Subjects were evaluated with Mini Mental State Examination (MMSE), Frontal Assessment Battery (FAB), and prose memory (Babcock story) tests. In addition, with functional Magnetic Resonance Imaging (fMRI), we investigated resting state network (RSN) activities. Resting state (Rs) fMRI data were analyzed by means of Independent Component Analysis (ICA). Subjects were followed-up with neuropsychological evaluations for three years.

**Results.** Rs-fMRI of MCI subjects showed increased intrinsic connectivity in the Default Mode Network (DMN) and in the Somatomotor Network (SMN). Analysis of the DMN showed statistically significant increased activation in the posterior cingulate cortex (PCC) and left inferior parietal lobule (lIPL). During the three years follow-up, 4 MCI subjects converted to AD. The subset of MCI AD-converted patients showed increased connectivity in the right Inferior Parietal Lobule (rIPL). As for SMN activity, MCI and MCI-AD converted groups showed increased level of connectivity in correspondence of the right Supramarginal Gyrus (rSG).

**Conclusions.** Our findings indicate alterations of DMN and SMN activity in MCI subjects, thereby providing potential imaging-based markers that can be helpful for the early diagnosis and monitoring of these patients.

## Introduction

Alzheimer disease (AD) is characterized by progressive neuronal degeneration that leads to deficit of cognitive functions and behavioral impairment. AD irreversibly damages neurons in critical brain circuits of the entorhinal cortex (EC), the thalamus, the hippocampus (Hp), and the limbic system (LS). AD is defined by the presence of two pathological hallmarks: intra and extra neuronal accumulation of beta amyloid (Aβ) and formation of neurofibrillary tangles that are aggregates of phosphorylated tau protein.

AD is a complex syndrome. In recent years it has become clear that the disease can manifest itself with a pleiotropic array of symptoms (Frautschy & Cole, 2010). Irrespectively from the initial clinical pattern of presentation, patients eventually move from a state of almost complete normality to severe cognitive deficits in the span of few years. Most of the times, patients initially experience mild memory or attention loss, deficits that have no major impact on daily routines. Deficits progress and, when the patient cognitive reserve is exhausted, eventually severely hamper their quality of life. The transitional period expanding from aging-related cognitive decline and early signs of AD is known as Mild Cognitive Impairment (MCI).

MCI is diagnosed when: (i) there is evidence of significant memory impairment but the patient’s general cognitive and functional abilities are still preserved and (ii) there are no sufficient diagnostic criteria to pose an alternative diagnosis of non-AD type of dementia. MCI patients can show a wide variety of symptoms. In that respect, MCI has been sub-categorized as amnestic MCI (aMCI), multiple cognitive domain MCI, and MCI without amnesia (non aMCI). Memory loss is a main feature and thought to be a high risk factor for the subsequent development of AD within few years (Jessen et al., in press).

Functional magnetic resonance imaging (fMRI) is a useful tool to investigate modifications in functional connectivity that, at least in part, may reflect changes in brain plasticity (Boyke et al., 2008; Lewis et al., 2009). fMRI also allows the evaluation of brain changes that occur during the progression from healthy aging to AD and can be used to identify individuals in the pre-symptomatic stages (Greene & Killiany, 2010). We chose to employ resting state fMRI (rs-fMRI) to study effects on functional connectivity. Compared to task-related fMRI, rs-FMRI offers some advantages. One of the major benefits is the potential to reduce confounding factors like inter-individual variability in task compliance and/or performance during fMRI acquisition (Ferreira & Busatto, 2013). rs-fMRI is also easier to employ in subjects affected by cognitive deficits.

When using fMRI, it is helpful to evaluate low-frequency fluctuations of cerebral hemodynamics (around 0.01–0.1 Hz). These fluctuations exhibit a complex spatial structure reminiscent of fMRI ‘activation maps’ and can be studied in rest conditions or upon the execution of tasks or external stimulations (Mantini et al., 2007).

In recent years, the characterization of these maps and the regional identification of slow variations in blood-oxygen level dependent (BOLD) signals have gathered considerable interest within the neuroimaging community. Many studies have suggested that these variations are of neuronal origin, temporally correlated across the brain, and correspond to functional resting-state networks (RSNs) (Sheline & Raichle, in press). These activities are thought to represent the neuronal baseline activity of the human brain in the absence of deliberate and/or externally stimulated activity and identify the presence of functionally distinct networks (Damoiseaux et al., 2006; Deco, Jirsa & McIntosh, 2011; Smith et al., 2009). The evaluation of brain resting state (RS) activity with fMRI can be helpful in studies aimed at investigating brain changes associated with pre-clinical dementia (Broyd et al., 2009; Mintun et al., 2006). Compared to task-related fMRI, rs-fMRI offers some advantages. Rs-fMRI allows the simultaneous investigation of multiple cortical circuits at once.

Using fMRI, many studies have extensively investigated functioning and anatomical correlates of the Default mode network (DMN), a system that includes the Medial Prefrontal Cortex (MPFC), the Posterior Cingulate Cortex (PCC), the Inferior Parietal Lobule (IPL), and the Hp (Buckner, Andrews-Hanna & Schacter, 2008). This network has been associated with reflective activity and self-referential mental processes (Broyd et al., 2009) and is employed to evaluate changes in functional connectivity occurring upon physiopathological conditions (Broyd et al., 2009; De Vogelaere et al., 2012; Sperling et al., 2009).

Within all the RSNs, DMN has received the greatest attention because it contains several regions that support cognitive functions and undergo critical changes upon aging as well as in neurodegenerative diseases including AD (Zhu et al., 2013). For that reason, we decided to investigate the DMN along with other major RSNs.

The identification of MCI patients is still mostly based on neuropsychological evaluations while no major functional markers are so far available. More importantly, no major neuroimaging markers have been identified to successfully predict who, in a cohort of MCI patients, is set to develop AD.

In the quest for novel neuroimaging marker, we here evaluated morpho-functional changes occurring in the brain of MCI and healthy elderly subjects investigated with rs-fMRI. The study also analyzed the same imaging parameters in MCI individuals that eventually developed AD.

## Materials and Methods

### Study population

The study was approved by the Institutional and Ethics Committee of the University “G. d’Annunzio” Chieti-Pescara (ID#157801). All procedures were conducted in accordance with principles expressed in the Helsinki Declaration. All study subjects gave written informed consent. Thirty seven volunteers (age ± SD: 60–80 y.o. ± 5.69) with comparable levels of education (8–10 years) were recruited. Subjects were initially screened through a careful neurological examination to exclude individuals showing visual and motor impairments, major medical conditions, psychiatric (confirmed by the Millon test) or neurological disorders and subjects taking psychotropic drugs. Physical and psychological examinations were performed and data recorded with particular focus on medical comorbidity. Examinations were conducted by trained psychologists and AD specialists (neurologists and psychiatrists).

### Genotyping

All study subjects gave written informed consent for collecting genomic DNA for genetic analysis. PCR amplification followed by direct DNA sequencing (ABI 3130xl genetic analyzer Life Technologies) were used to determine APOE genotypes (APOE 2, 3, 4) associated with single nucleotide polymorphisms (Rs 7412, Rs 429358; Sun et al., 2011). DNA was extracted from buccal brushes using the Nucleo Spin Tissue kit (M-Medical).

### Neuropsychological assessment

Subjects were selected and assessed with the following neuropsychological tests: Mini Mental State Examination (MMSE) to evaluate the global cognitive status; prose memory test (Babcock story) to evaluate prose memory, and the Frontal Assessment Battery (FAB) to screen for global executive functions. With the MMSE, we divided subjects in two groups: 13 subjects with a score ranging between 21 and 25 were identified as the MCI group while 24 elderly subjects with a 26–30 score were considered as the healthy control (HC) group (in accordance with criteria described in Iliffe et al., 1990). Study subjects were followed-up with neuropsychological evaluations and tested three years after the first evaluation. In that time frame, we identified 4 subjects who converted from MCI to AD (MCI AD-converted); of these four patients, two subjects died because of AD-related complications.

### Rs-fMRI Acquisition

Functional and structural fMRI imaging was performed with a Philips Achieva 3T Scanner (Philips Medical Systems, Best, The Netherlands) using a whole-body radiofrequency coil for signal excitation and an eight-channel head coil for signal reception. BOLD fMRI data were acquired in four runs lasting four minutes each by means T2*-weighted echo planar imaging (EPI) free induction decay (FID) sequences applying the following parameters: TE 35 ms, matrix size 64 × 64, FOV 256 mm, in-plane voxel size 4 × 4 mm, flip angle 75°, slice thickness 4 mm and no gap. Functional volumes consisted of 30 trans-axial slices, acquired with a volume TR of 1671 ms. A high resolution structural volume was acquired at the end of the session via a 3D fast field echo T1-weighted sequence (sagittal, matrix 256 × 256, FOV 256 mm, slice thickness 1 mm, no gap, in-plane voxel size 1 mm × 1 mm, flip angle 12°, TR = 9.7 ms and TE = 4 ms). Subjects were asked to relax while fixating the center point of a grey-background screen projected via an LCD projector and viewed via a mirror placed above the subject’s head.

### Data analysis

**Figure 1:**
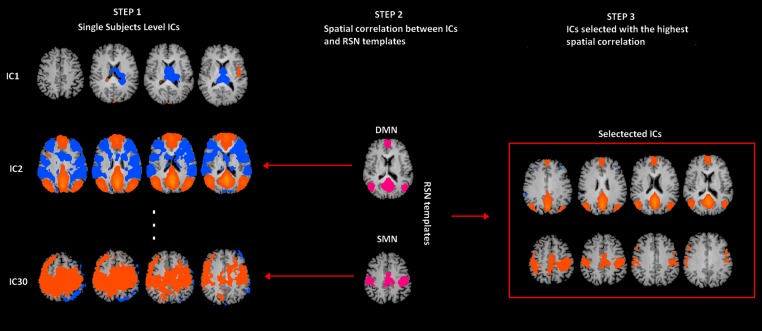
rs-fMRI processing. Selection of two Resting State Networks among the independent components (ICs) were obtained by means of the Fast-ICA algorithm implemented in Brain Voyager QX. In Step 1, individual single subject IC maps were obtained (only three components are depicted). In Step 2, the map of each component (only three components depicted) is spatially correlated with a network template (only for SMN and DMN). Finally, in Step 3, the component with the largest spatial correlation coefficient is selected. SMN and DMN are from a previous study (Mantini et al., 2007).

BOLD fMRI data were analyzed by means of the Brain Voyager QX 1.9 software (Brain Innovation, The Netherlands). Due to T1 saturation effects, the first 5 scans of each run were discarded from analysis. Pre-processing of functional scans included motion correction, removal of linear trends from voxel time series and slice scan-time correction. To match each functional volume to the reference volume, the motion correction was performed by three-dimensional rigid body transformation. The estimated translation and rotation parameters for each volume in the time course were inspected to check that movements were not larger than approximately half a voxel (Friston et al., 1996; Hajnal et al., 1994). Pre-processed functional volumes of single subjects were co-registered with the corresponding structural data set. 2D functional and 3D structural measurements were acquired in the same session and therefore the co-registration transformation was determined using the position parameters of the structural volume. The alignment between functional and anatomical scans was finally checked by means of accurate visual inspections. Structural and functional volumes were transformed into Talairach space using a piecewise affine and continuous transformation. Functional volumes were re-sampled at a voxel size of 3 mm × 3 mm × 3 mm. Two covariates that modeled signals sampled from White Matter (WM), Cerebro-Spinal Fluid (CSF) were included in the analyses (Fox & Raichle, 2007; Weissenbacher et al., 2009). We derived WM and CSF signals by averaging time courses of voxels in each subject WM masks and CSF. WM masks were generated by the segmentation process of each subject brain, while CFS signals were sampled from the third ventricle of each subject brain. Spatial independent component (IC) analysis was used to analyze rs-fMRI data sets for the decomposition of voxel time series into a set of independent spatiotemporal patterns (ICs) (McKeown et al., 1998). At first, a single subject Independent Component Analysis (ICA) was performed, separately for each of the four runs, using a plugin extension of BrainVoyager QX based on the FastICA algorithm (Hyvarinen, 1999). 30 ICs were extracted for each data set and scaled to spatial *z*-score maps with a deflation approach and Tahn nonlinearity. The time course of each IC is the waveform of a specific pattern of coherent brain activity. The intensity of this pattern is expressed in the corresponding spatial map (Mantini et al., 2007). By removing the average value and dividing by the standard deviation of the intensity distribution, intensity values in each spatial map were converted to *Z* values. It is commonly accepted that *Z* values, obtained from individual maps, provide an indirect measure of functional connectivity within a selected network (Calhoun et al., 2001; Liao et al., 2010). IC spatial maps were scaled to *z*-scores to allow comparisons across sessions and subjects. In each IC map, the *z*-score value that is associated to a given voxel reflects the weight of IC time course with respect to the relative measured BOLD data, thereby providing an indirect indication of functional connectivity (McKeown et al., 1998). After exclusion of artefactual ICs based on the IC-fingerprint method (De Martino et al., 2007), we selected ICs showing the largest spatial correlation with RSN templates obtained in a previous resting-state study conducted on healthy volunteers (Mantini et al., 2007). This approach is in line with previous resting-state studies and assumes that there is a canonical spatial pattern that allows a reliable detection at the single-subject level using a template-matching procedure (Mantini et al., 2007; Fig. 1). Later on, ICs from the four within-subject data sets were clustered using the self-organizing group-level ICA (sog-ICA) algorithm (Esposito et al., 2005) that is implemented in BrainVoyager QX with the creation of a single subject IC data set (within-subject analysis). Sog-ICA was then applied to clusters within subject data sets (across subject – analysis) (Mantini et al., 2009). Regions of interest (ROIs) were created from these RSN templates and employed to compute between-group differences. For each RSN, between-group differences were assessed by means of a voxel-wise one way ANOVA on *Z* values [*p* < 0.05; Bonferroni corrected] obtained from individual ICA group maps. Clusters of interest were considered only when included in nodes of each IC of interest. After voxel-wise analysis, *Z* values from clusters showing a between-group difference were extrapolated and a two-tailed t test was performed.

## Results

### rs-fMRI evaluation

**Figure 2:**
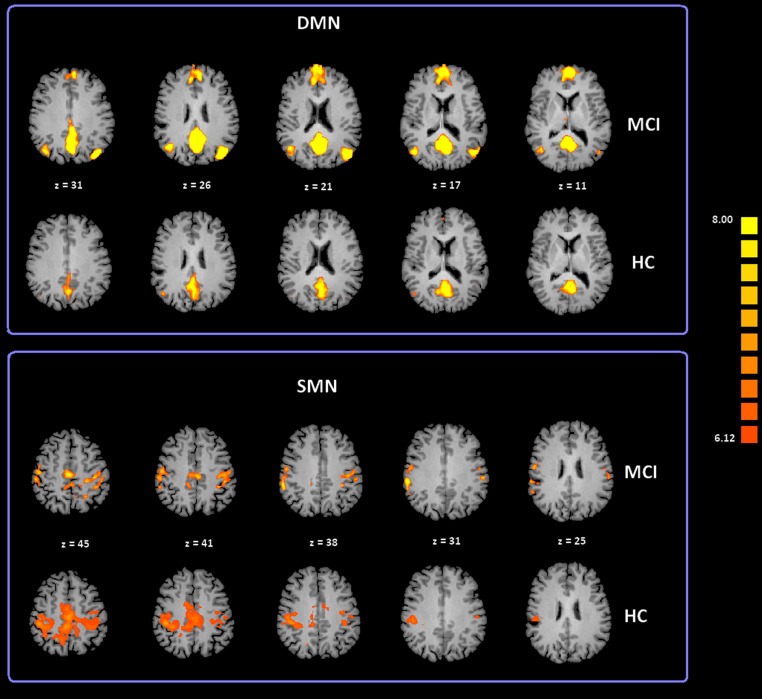
Cortical representation of two group level RSNs (DMN and SMN) in MCI patients and HC. Figure depicts transverse views of the brain for each group. RSN maps are overimposed on a Talairach template. Color scale represents T values.

To date, at least ten RSNs have been identified (Damoiseaux et al., 2006; Deco, Jirsa & McIntosh, 2011; Mantini et al., 2009; van den Heuvel & Hulshoff Pol, 2010). Of these ten, the most studied include: the DMN; the Salience Network (SN); the Fronto Parietal Control (FPC) network (lateralized in both hemispheres); the primary Sensory Motor Network (SMN), the Exstrastriate Visual System (EsV), and the Dorsal Attention Network (DAN). In our study we analyzed all these ten RSNs but only the DMN and SMN showed significant differences between subject groups. ICA group classification revealed a typical spatial pattern for DMN and SMN in the MCI and HC groups. Our procedure for ICA classification produced consistent DMN and SMN maps as illustrated in Fig. 2 and Table S1 provides a list of the brain regions associated with each network, along with Talairach coordinates of the mean peaks foci and the associated Brodmann areas (BA). Two-tailed t-test revealed differences in DMN and SMN functional connectivity between two groups. The MCI group showed significant increased *Z* values for the DMN with *t*(35) = 3.02 and *p* < 0.01 corrected for multiple comparisons. Moreover, the MCI-AD converted group showed significant increased *Z* values for the DMN with *t*(26) = 2.60 and *p* < 0.05 corrected for multiple comparisons. In the case of the SMN, the MCI-AD converted group showed significant increased *z* values when compared to the MCI group (*t*(11) = 2.43; *p* < 0.05 uncorrected) or the HC group (*t*(26) = 3.78; *p* < 0.005 corrected for multiple comparisons).

A voxel wise one-way ANOVA was performed with the BrainVoyager ANOVA tool to evaluate DMN and SMN maps. In the case of the DMN, significant increased values of connectivity were observed in the PCC and left IPL (lIPL) for the MCI group when compared to the HC group. In the MCI-AD converted group comparison to HC by ANOVA showed significantly increased connectivity values in the right IPL (rIPL).

**Figure 3:**
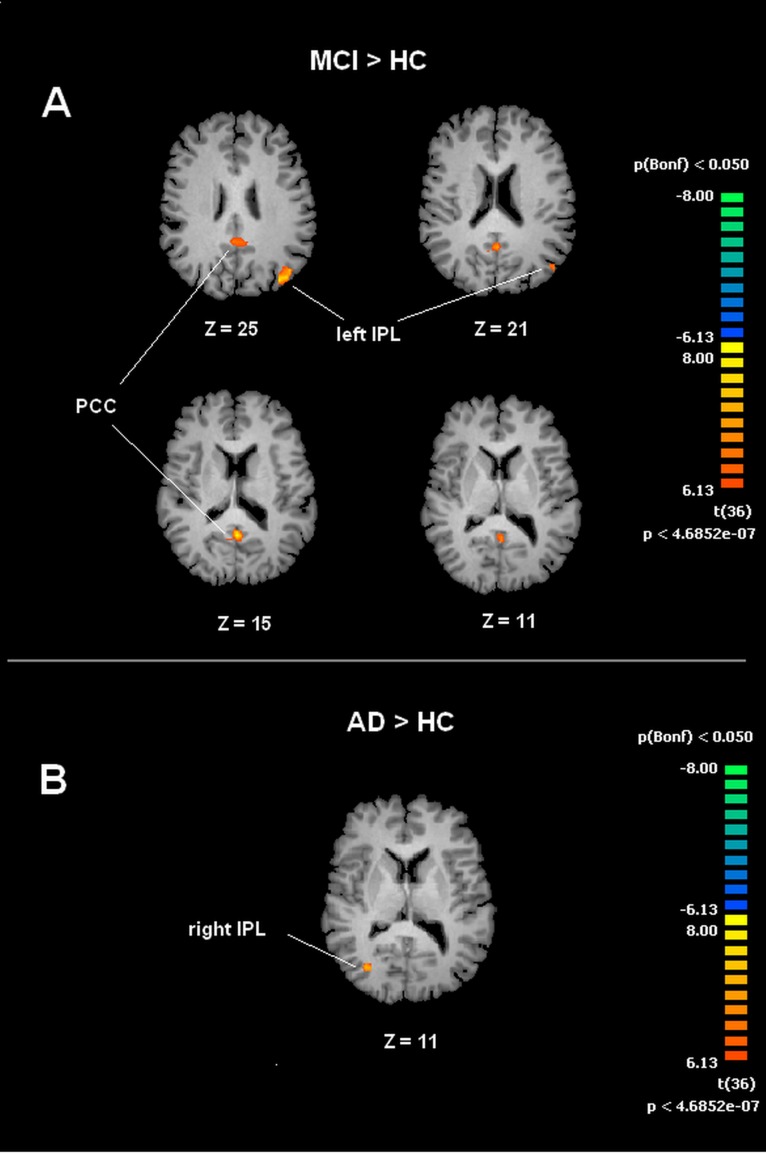
Between group differences in DMN for MCI patients and Healthy controls (HC). Panel (A) shows t-maps obtained when comparing MCI and HC. T-test comparisons reveal statistically significant increased levels of intrinsic connectivity in the Posterior Cingulated Cortex (PCC) and left Inferior Parietal Lobe (left IPL) in the MCI group. Panel (B) shows t-maps obtained when comparing MCI-AD converted and HC. Between group comparisons show significant increased values of intrinsic connectivity in the right Inferior Parietal Lobe (right IPL) in MCI-AD converted group. Functional maps shown in A and B are Bonferroni corrected (*p* < 0.05) and overimposed on a Talairach template.

**Figure 4:**
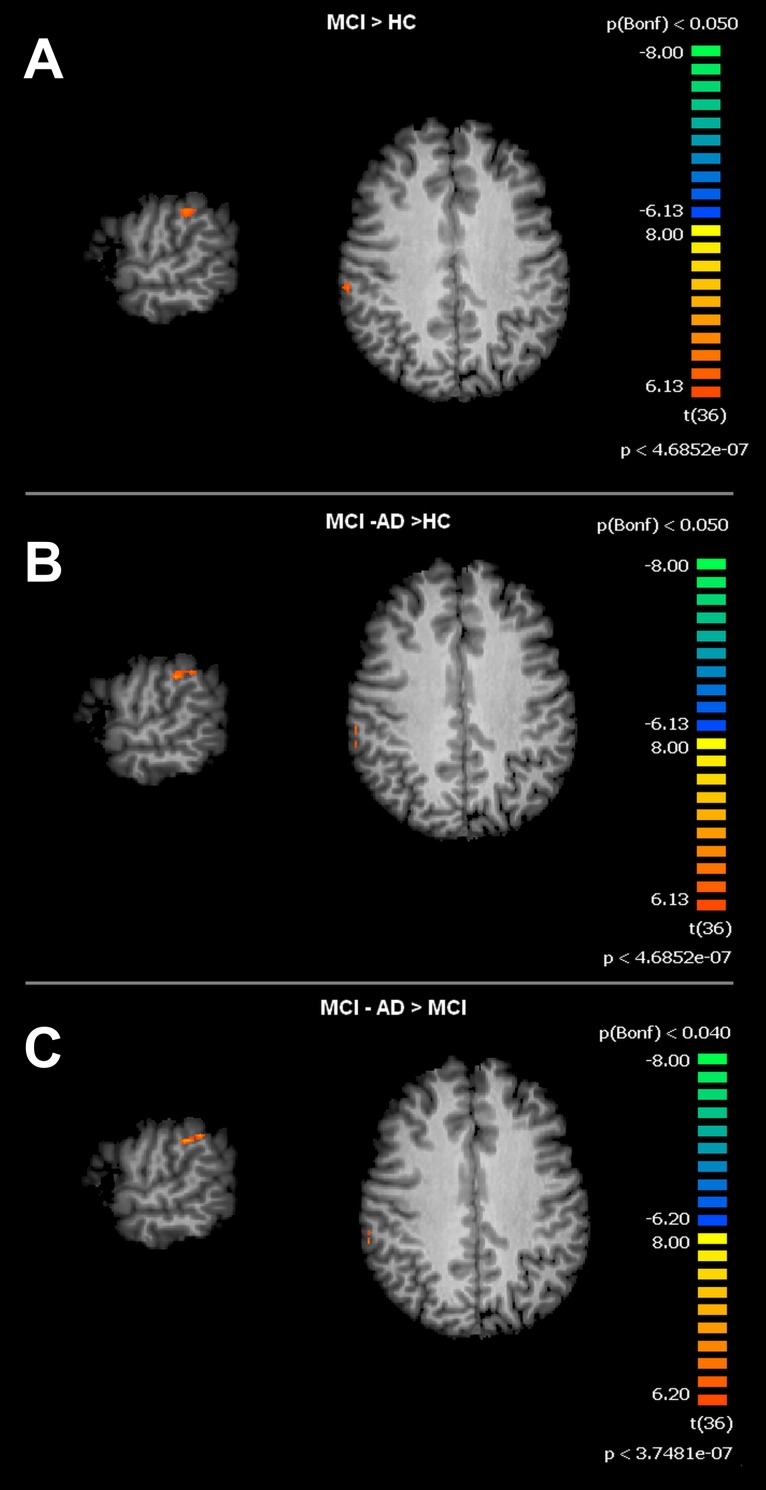
Between group differences in SMN for MCI patients and Healthy controls (HC). Figure depicts t-maps obtained when comparing MCI and HC (A), MCI-AD converted and HC (B) or MCI-AD converted and MCI (C). T-test comparisons reveal statistically significant increased levels of intrinsic connectivity in the right supramarginal gyrus for the contrast MCI > HC, MCI-AD > HC and MCI-AD > MCI. Functional maps are Bonferroni corrected (*p* < 0.05) and overimposed on a Talairach template.

For the SMN, the MCI group, when compared to HC, showed significant increased connectivity in the right Supramarginal Gyrus (rSG). The MCI-AD converted group showed increased levels of connectivity of the rSG compared to either the MCI or HC groups. Voxel-wise ANOVA results are shown in Figs. 3 and 4.

### APOE genotyping

Genotype analysis showed that MCI and HC were mostly (80%) carrying the 3/3 genotype with only two MCI subjects carrying the 3/4 genotype. For the MCI AD-converted group we were able to genotype only one subject and he was 3/3.

## Discussion

The implementation of the concept of normality while studying brain aging is not always easy. This is related to the intrinsic grey zone that spans between physiological and pathological when considering the aging process. Morpho-functional changes occurring in the aging brain are sometimes prodromal to degenerative evolutions but can also be part of compensatory mechanisms (Deco, Jirsa & McIntosh, 2011; Pieramico et al., 2012).

It should be also emphasized that brain aging and AD are deeply interconnected (Kovacs, Cairns & Lantos, 1999). AD deeply affects citizen health as well as the wealth of public health systems and, with the growing rate of elderly people in western countries, is becoming a health/economic issue of epidemic proportions (Hurd et al., 2013). A recent, intriguing, and promising approach in the field of AD concerns the very early use of imaging and biological markers to identify at risk subjects decades before the manifestation of clinical signs of the disease (Holtzman, Mandelkow & Selkoe, 2012; Jack et al., 2013).

Many studies (Fleisher et al., 2009; Mintun et al., 2006; Sperling et al., 2009) have provided strong evidence supporting the idea that identifiable changes in brain physiology occur prior to the appearance of clinical signs of AD. fMRI offers the possibility to investigate dynamic changes in brain activity occurring in prodromal phases of AD. In that respect, pre-clinical AD has been associated with early detection of pathological modifications involving retrosplenial regions at first and then spreading further to the Hp, perirhinal cortex (PC), EC, LS, and the orbitofrontal cortex (OFC) (De Vogelaere et al., 2012). Most of these regions are involved in RSNs. Of all the RSNs, the DMN has received the greatest attention because it contains several regions that undergo critical changes upon aging and AD (Buckner, Andrews-Hanna & Schacter, 2008; Zhu et al., 2013)

Recent studies have investigated another important network, known as SMN, which has been indicated to be involved in several neurological or psychiatric conditions (AD, schizophrenia or depression). Within the SMN, the inferior parietal cortex (IPC) and the SG play an important role in the modulation of episodic memory (Liang et al., 2013).

In our study, we evaluated thirty seven subjects with a battery of neuropsychological tests and fMRI scans in order to identify potential functional biomarkers in the early stage of the disease. Differences in DMN and SMN intrinsic connectivity in the two groups were observed.

Our fMRI results indicate a rearrangement of the DMN that results in enhanced intrinsic connectivity in the PCC of MCI subjects. Increased DMN connectivity has been shown to occur in degenerative diseases (AD, multiple sclerosis) and neuropsychiatric conditions like attention deficit hyperactivity disorder (ADHD), schizophrenia, and autism (Broyd et al., 2009).

The PCC is the most common site of early metabolic and perfusion abnormalities occurring upon aging and AD (Buckner, Andrews-Hanna & Schacter, 2008; Chetelat et al., 2003). Disrupted connectivity between the Hp/EC and the PCC has been proposed as a functional mechanism of PCC hypometabolism and hypoperfusion, phenomena that are observed in the early stage of AD (Mevel et al., 2011). The PCC has been also identified as the region showing early Aβ deposition in elderly individuals and in AD patients (Koch et al., 2010).

The increased PCC connectivity that we observed in MCI subjects can be interpreted as a compensatory mechanism to counteract neuronal dysfunction associated with subthreshold Aβ accumulation in a region that is a major hub for memory circuits (Qi et al., 2010). Moreover, our fMRI data show significant increase of intrinsic connectivity in the lIPL in the MCI group and in the rIPL of AD-converted MCI patients. To date, little is known on how the IPL can be affected during the progression to AD. However, changes in the region have been reported and interpreted as potential functional AD markers (Greene & Killiany, 2010).

The IPL is positioned between the SG, the lateral occipital cortex (LOC), the superior parietal gyrus (SPG), and the middle temporal gyrus (MTG). The region is a sensory motor associative area. Autopsy studies on MCI and AD patients have shown the presence of Aβ buildup in the IPL (Nelson et al., 2009). Studies on animal models have demonstrated anatomical connections between the IPL and temporomesial regions (TMR) like the Hp and EC (Ding, Van Hoesen & Rockland, 2000), thereby supporting the idea that the IPL may have a major role in modulating memory functions (Sestieri et al., 2013). Changes between left and right in the increased IPL intrinsic connectivity that we observed among MCI and AD-converted MCI patients can be hypothesized as an indication of a trend toward shifting IPL laterality in pathological conditions.

Our data also show significant differences between healthy elderly and MCI subjects in terms of lIPL connectivity and are in line with previous reports (Greene & Killiany, 2010).

It is interesting to note the increased rIPL connectivity that emerges from a retrospective analysis of fMRI data on the subgroup of MCI patients who, in three years, converted to AD. Evidence indicates that the rIPL may become affected in MCI subjects that are more prone to convert to AD (Greene & Killiany, 2010). Our data are in line with these previous findings (Greene & Killiany, 2010; Turner & Spreng, 2012).

The SMN, including the right SG, plays an important role in episodic memory, action recognition and spatial navigation (Russ et al., 2003). The increased intrinsic SMN connectivity that we observed in the MCI and MCI AD-converted groups may be interpreted as a compensatory mechanism set in motion in the attempt to counteract cognitive decline.

The increased SG connectivity that we found in the MCI AD-converted group compared to the MCI group has the potential to be considered a neuroimaging marker that could help in predicting AD conversion (Hayes, Salat & Verfaellie, 2012; Liang et al., 2013). Our results show a significant between-group difference for the SMN with increased values in correspondence of SG. This brain region seems to be related to sensory/attentional components of action programming and, at the same time, is involved in the perception of action (Hartwigsen et al., 2012; Medina et al., 2009).

Moreover, the Angular Gyrus (AG) and SG are implicated in viewer-centered (egocentric) processing during spatial exploration tasks. Subjects affected by MCI or AD are known to experience difficulties with spatial navigation (Nedelska et al., 2012) and have signs of topographical disorientation (Pai & Jacobs, 2004).

Data indicate that upon spatial navigation tasks, MCI individuals who are at higher risk for AD prefer egocentric strategies and the phenomenon has been interpreted as an index of cognitive decline (Laczo et al., 2011).

AD and MCI subjects show increased brain atrophy in several regions like the right Hp, the superior temporal gyrus (STG) and MTG, the inferior frontal gyrus, the inferior parietal gyrus (IPG), and the inferior supramarginal gyrus (ISG).

The increased intrinsic connectivity within SG that we found in the MCI and MCI AD-converted subjects may therefore represent a mechanism aimed at counteracting brain atrophy of these regions and reducing the associated cognitive decline.

In that respect, decreased medial temporal lobe (MTLE) activity can be related to underlying memory impairments of MCI subjects (Larocque et al., 2013).

The increased intrinsic SG connectivity that we found in MCI patients suggests compensatory processes set in motion by initial cognitive deficits (Prvulovic et al., 2002). The compensatory hypothesis has been postulated for MCI and AD patients (Bookheimer et al., 2000). The increased activity can be read as an attempt by MCI patients to overwork network resources, primarily in the SG and IPL regions, in order to maintain memory functions.

Limitations of this study include the heterogeneity and the consequent difficulty to subdivide MCI in different categories as aMCI and non-aMCI, a problem linked to our small sample size. Also because of the limitations imposed by our sample size, no direct correlations could be drawn between the likelihood to progress to AD and the expression of specific APOE genotypes.

In conclusion, our data lend some support to the idea that MCI subjects can compensate for degenerative processes by activating a residual neuronal plasticity that reorganizes functional networks, thereby delaying the expression of clinical signs of frank AD.

Our study also underlines the importance of combining neuropsychological and neuroimaging approaches to study early stages of AD as well as transitional stages occurring from MCI to AD.

A better knowledge of these pathophysiological steps can help to establish pharmacological and non-pharmacological interventions aimed at maximizing the patient cognitive reserve and extend the duration of the preclinical phase.

## Supplemental Information

10.7717/peerj.135/supp-1 Table S1 Brain areas relative to DMN and SMN of the three study groups (HC, MCI, and MCI-AD)
