# Supplementary material for: Characterization of resting state activity in MCI individuals
Source: PeerJ. 2013 Aug 20;1:e135. doi: 10.7717/peerj.135 (PMC3757508; doi:10.7717/peerj.135)
Supplement: Table S1 [file peerj-01-135-s001.docx]

| **Default Mode Network** | | | | | | |
| --- | --- | --- | --- | --- | --- | --- |
| **Cluster** | **Hemisphere** | **BA** | **X** | **Y** | **Z** | **t** |
| InferiorParietalLobe | R | 39 | 47 | -65 | 15 | 12,100386 |
| PosteriorCingulateCortex / Retrosplenial | L | 29 | -1 | -53 | 15 | 18,588511 |
| InferiorParietalLobe | L | 39 | -46 | -68 | 21 | 14,620455 |
| VentromedialPrefrontalCortex | R | 10/32 | 2 | 49 | 39 | 12,86483 |
|  | **MCI >HC** | | | | | |
|  | **Hemisphere** | **BA** | **X** | **Y** | **Z** | **t** |
| PosteriorCingulateCortex | L | 29 | -1 | -53 | 15 | 8,192981 |
| InferiorParietalLobe | L | 39 | -37 | -77 | 27 | 8,906489 |
|  | **MCI AD converted> HC** | | | | | |
|  | **Hemisphere** | **BA** | **X** | **Y** | **Z** | **t** |
| InferiorParietalLobe | L | 39 | 29 | -68 | 12 | 7,71915 |
| **Somatomotor Network** | | | | | | |
|  | **Hemisphere** | **BA** | **X** | **Y** | **Z** | **t** |
| SupramarginalGyrus | R | 40 | 59 | -29 | 33 | 10,29 |
| PrecentralGyrus | R | 4 | 47 | -17 | 42 | 8,2 |
| AnteriorParietalLobe | R | 5 | 35 | -41 | 57 | 8,4 |
| MedialFrontalGyrus /DorsalCingulateGyrus | R | 4/24 | 2 | -17 | 45 | 10,45 |
| SuperiorParietalLobe/SupramarginalGyrus | L | 7/40 | -22 | -44 | 57 | 9,48 |

*Brain areas relative to DMN and SMN of the three study groups (HC, MCI, and MCI-AD)*

Brain regions are listed in accordance with Talairach coordinates (x: left-right; y: anterior-posterior; z: dorsal ventral) of the peaks of clusters and the corresponding t and p<0.05 Bonferroni corrected. Abbreviations: BA: Brodmann’s area; L: left; R: right
